# Successful Management of a Ruptured Interstitial Pregnancy: A Case Report

**DOI:** 10.7759/cureus.39377

**Published:** 2023-05-23

**Authors:** Laila Y Alhubaishi, Anjala Nizam, Sofia A Malik

**Affiliations:** 1 Department of Obstetrics and Gynaecology, Latifa Hospital, Dubai, ARE; 2 Department of Medicine and Surgery, Dubai Academic Health Corporation, Dubai, ARE

**Keywords:** cornual excision, cornuostomy, ectopic pregnancy, interstitial pregnancy, uterine rupture

## Abstract

Interstitial pregnancy is a rare entity that usually leads to the rupture of the uterus. The difficulty and delay in diagnosing this condition have been reported to cause high mortality rates. Here, we present the case of a 36-year-old woman who presented to the emergency department with severe epigastric pain and hemodynamic instability. Her current gestational age (GA) could not be accurately measured as she was unbooked and had irregular periods. However, by abdominal examination, the GA was estimated to be 38 weeks, whereas by ultrasound it was approximately 28 weeks. It was also noted that the uterus was empty, and the fetus was found in the abdominal cavity. Hence, a working diagnosis of uterine rupture was made and the patient was taken for emergency laparotomy. The patient delivered an alive 1.2 kg baby. Intraoperatively, the placenta was implanted in the interstitial part of the right fallopian tube. The placenta was then excised and right salpingectomy was performed, following which the abdomen was closed in layers. Postoperatively, the patient made an uneventful recovery and was discharged home in stable condition; however, the baby passed away due to complications related to extreme prematurity.

## Introduction

The term interstitial ectopic pregnancy refers to an ectopic pregnancy that occurs in the uterine portion of the fallopian tubes. It is a rare type of ectopic pregnancy responsible for only 5% of all tubal pregnancies [1]. A delayed diagnosis and the high vascularity of the myometrium make it more likely to cause shock and hemoperitoneum following its rupture than any other forms of ectopic pregnancy [2]. Here, we present the case of a 36-year-old female who had a ruptured right interstitial pregnancy and presented to the emergency department (ED) in shock.

## Case presentation

A 36-year-old woman, G4P3+0 with an unknown last menstrual period due to irregular menstrual cycles, presented to the ED with severe epigastric pain that started a few hours ago along with dizziness. Her first two children were delivered through normal vaginal delivery, whereas her last delivery was by lower-segment cesarean section approximately two years ago. In the current pregnancy, she received insufficient antenatal care and was considered unbooked. Upon arrival, she was conscious and oriented; however, she was in shock, with marked hypotension and blood pressure of 50/22 mmHg. Other vital signs were within the normal range with a heart rate of 85 beats per minute, respiratory rate of 18 breaths per minute, temperature of 37.3°C, and oxygen saturation of 98%. On physical examination, she appeared severely pale, and abdominal examination was remarkable for abdominal tenderness and rigidity all over. On obstetric examination, her abdomen was approximately 38 weeks in size. A bedside transabdominal ultrasound scan revealed an empty uterus, a fetus of approximately 28 weeks gestational age in the abdominal cavity with positive fetal cardiac activity, and blood clots all over the abdominal cavity. A working diagnosis of uterine rupture was made and immediate resuscitative measures were started with intravenous fluids and Haemaccel (Piramal Enterprises, India). Her labs were sent while pushing her to the operation theater for emergency exploratory laparotomy (Table 1).

**Table 1:** Laboratory investigations. WBC: white blood cell; RBC: red blood cell; INR: international normalized ratio; APTT: activated partial thromboplastin time; pCO_2_: partial pressure of carbon dioxide; pO_2_: partial pressure of oxygen; HCO_3_: bicarbonate

Laboratory investigation	Laboratory value	Normal reference range
Complete blood count		
WBC count	13.3 k/µL	3.6–11 k/µL
RBC count	3.31 MIL/µL	3.80–4.80 MIL/µL
Hemoglobin, blood	7.9 g/dL	12–15 g/dL
Hematocrit	25.0%	36%–46%
Platelet count	246 k/µL	150–410 k/µL
Coagulation profile		
Prothrombin time	13.4 seconds	11.5–14.5 seconds
INR	1.04	0.80–1.20
APTT	33.8 seconds	28.6–38.2 seconds
Fibrinogen	293 mg/dL	190–430 mg/dL
Arterial blood gas metabolic panel		
pH	7.274	7.35–7.45
pCO_2_	36.7 mmHg	35–45 mmHg
pO_2_	185 mmHg	83–108 mmHg
HCO_3_	16.4 mmol/L	21–28 mmol/L
Lactate	2.4 mmol/L	0.5–1.6 mmol/L

**Figure 1:**
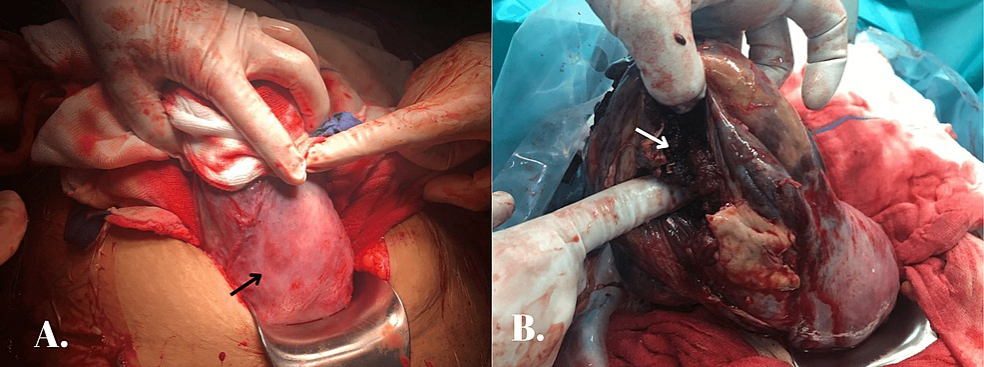
(A) Intact lower segment of the uterus (black arrow). (B) Placenta (white arrow) implanted in the interstitial portion of the right fallopian tube.

Intraoperatively, she received blood products including four units of packed red blood cells and two units each of fresh frozen plasma and platelets. The decision-to-delivery interval was approximately 35 minutes. Her intraoperative findings included a hemoperitoneum of 2 L, and the baby was found within the abdominal cavity and delivered. The right side of the fundus of the uterus was found to be ruptured; however, the lower segment of the uterus was found to be intact (Figure 1A). A normal-looking placenta was found implanted in the interstitial region of the right fallopian tube (Figure 1B) with adhesions to the posterior wall of the uterus, omentum, and bowels.

**Figure 2:**
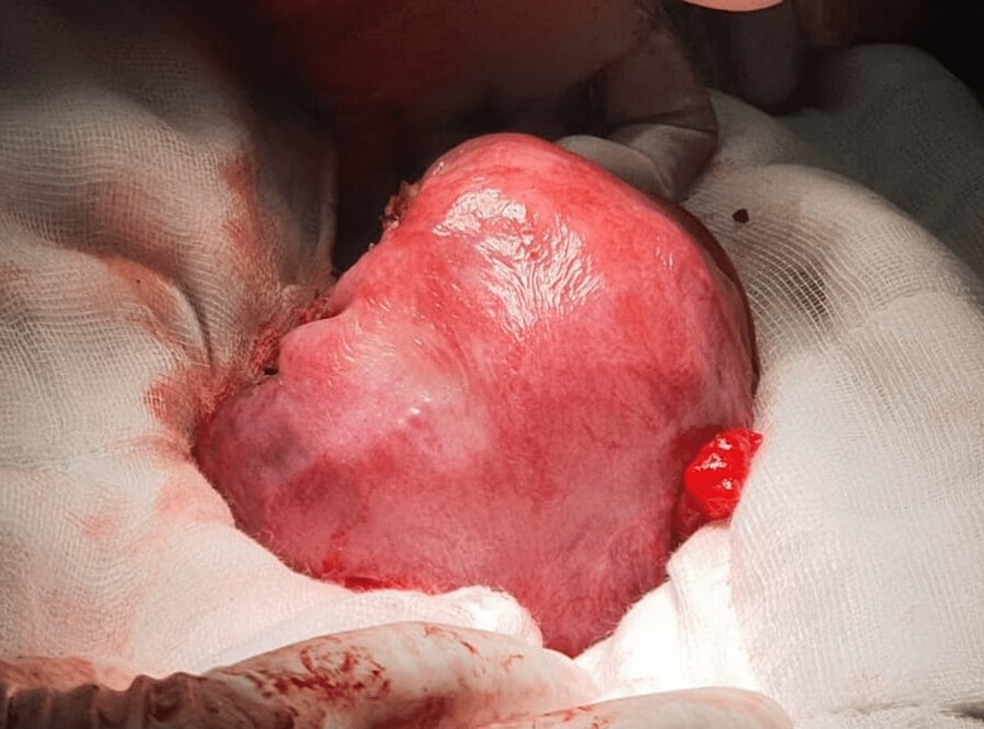
The uterus after correction and closure.

The placenta was delivered completely, adhesions were released, and a right-sided salpingectomy was done, following which the right cornua was closed in three layers (Figure 2).

**Figure 3:**
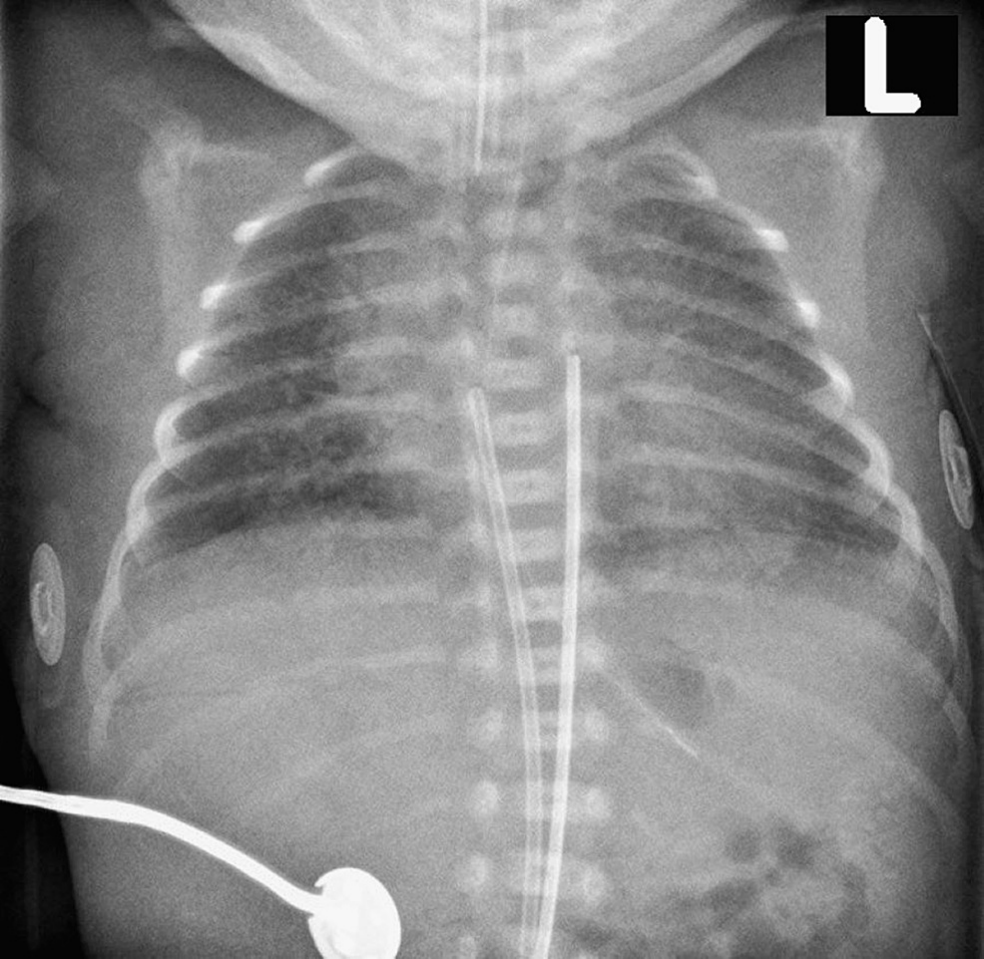
X-ray of the baby showing bilateral perihilar haziness.

The procedure went uneventful and her postoperative recovery was at a normal pace. The baby was a female born alive and weighed 1.2 kg. She was dusky in color upon birth and was started on positive-pressure ventilation immediately after. However, because oxygen saturation did not increase above 80%, the baby was nasally intubated and shifted to the neonatal intensive care unit. An X-ray was done which showed bilateral perihilar haziness due to neonatal respiratory distress syndrome (Figure 3).

The baby had several desaturation and bradycardic episodes; hence, she received cardiopulmonary resuscitation and was started on ionotropic support with dopamine, dobutamine, and epinephrine. Unfortunately, the baby passed away at 15 hours of life despite resuscitative measures, with the cause of death being lung hypoplasia due to extreme prematurity. The patient was discharged in stable condition on postoperative day three with follow-up advice to have effective contraception and abstain from any further pregnancy.

## Discussion

When a pregnancy is implanted in the proximal fallopian tube just as it enters the uterine myometrium, it is referred to as an interstitial pregnancy. This portion of the fallopian tube is 2 cm long, 0.7 mm broad, and tortuous in shape. It is bordered by a highly vascular myometrium and has the ability to grow, which could cause a rupture and cause severe hemorrhage [3]. The mortality rate for ruptured interstitial ectopic pregnancies is seven times greater than that of other ectopic pregnancies, making them an uncommon but potentially lethal event. Most often, the increased maternal mortality rate is attributed to the difficulty in diagnosis which results in delayed or missed diagnosis [4]. On ultrasonography, the following features are necessary for the diagnosis of interstitial ectopic pregnancy: (i) an empty uterine cavity; (ii) a gestational sac that is at least 1 cm from the lateral uterine wall; and (iii) a thin (5 mm) myometrial layer enclosing the gestational sac. These criteria only apply during the first trimester before the gestational sac enlarges. Given this, it is possible the patient mentioned in this case report could have been diagnosed with interstitial pregnancy and received treatment in a timely manner if she had been receiving appropriate antenatal care. The clinical diagnosis has a major role in ruptured intestinal pregnancy. Other symptoms including abdominal or pelvic discomfort, vaginal bleeding, shoulder pain, gastrointestinal symptoms, urinary symptoms, rectal pressure or pain on defecation, dizziness, fainting, and syncope are frequently present along with hypotension and tachycardia. On physical examination, the patient might have signs of hemoperitoneum, such as a rigid abdomen, as well as rebound abdominal tenderness, cervical motion tenderness, and/or abdominal distention. Pallor and an enlarged uterus, sometimes beyond the estimated gestational age, are further examination findings [4]. The decision of choice of management of intestinal pregnancy depends on the hospital’s facilities as well as the patient’s hemodynamic stability, gestational age, and desire for fertility. The method of choice for the medical management of interstitial pregnancy is an intramuscular injection of methotrexate [5]. Surgical management options include salpingectomy, salpingostomy, cornuostomy, and cornual resection, and vary on a case-to-case basis [6]. The standard approach for surgical management nowadays is laparoscopy. A fertility-sparing laparoscopic procedure that entails the excision of the interstitial segment of the tube (cornual resection) and evacuation of the interstitial pregnancy by cornuostomy should be taken into consideration in appropriate instances [7]. The patient mentioned in this study had completed her family; hence, a right-sided salpingectomy was performed along with cornual repair. Patients with hemodynamic impairment or situations where laparoscopic expertise is lacking typically require a laparotomy. If ongoing bleeding occurs and hemostasis cannot be achieved adequately during surgical management of interstitial pregnancy, a hysterectomy may be considered. Hysterectomies have been used to manage interstitial pregnancies at a rate as high as 40% [3]. The main complication of medically treated interstitial pregnancies is recurrence. Uterine rupture in subsequent pregnancies is most common with surgical approaches, especially by cornual resection, probably due to the fragility of the uterine wall [5].

A hemodynamically unstable pregnant patient with an ultrasound that suggests a ruptured uterus should undergo immediate surgery [8]. More recent studies support cornuostomy over cornual excision. A cornuostomy excises interstitial pregnancy while conserving uterine architecture and sustaining fertility. Cornuostomy is considered to cause less tubal damage than cornual resection and may have better pregnancy outcomes in the future along with the possibility of managing intestinal pregnancies using a minimally invasive approach, rather than open surgery [8]. A study by Tulandi et al. found that the mean volume of intra-abdominal hemorrhage encountered in a group of patients who underwent laparotomy was higher compared to patients who underwent laparoscopy [9]. However, this difference probably reflects the surgeon’s preoperative decision-making by taking into account the patients’ differing preoperative blood loss or hemodynamic stability, rather than blood loss caused by the operative technique itself.

## Conclusions

The major cause of high mortality and morbidity in interstitial pregnancy is delayed diagnosis and diagnostic dilemma, therefore, early diagnosis plays a major role in the prognosis. It is important to be aware of the unusual clinical presentation. Clinicians should be vigilant and keep ruptured interstitial pregnancy as a differential diagnosis in any pregnant woman who reports severe abdominal pain in the second or third trimester, especially those who have had poor antenatal care. Women with a history of interstitial pregnancy managed surgically must be counseled regarding risks associated with any future pregnancy. They can also be offered the option of having tubal ligation as a form of permanent contraception. In case of any future pregnancy, it should be labeled as a high-risk pregnancy and managed accordingly.
